# Daily consumption of sulfate- and magnesium-rich mineral water enhances bowel function in adults with chronic constipation: a randomized double-blind real-world trial

**DOI:** 10.3389/fnut.2026.1924028

**Published:** 2026-09-24

**Authors:** Katharina Mansouri, Thierry Hanh, Ronja Meyer, Andreas Hahn

**Affiliations:** 1 Institute of Food and One Health, Leibniz University Hanover, Hanover, Germany; 2 Independent Researcher, Paris, France

**Keywords:** bowel function, clinical trial, constipation, magnesium, mineral water, sulfate

## Abstract

**Introduction:**

Functional constipation is one of the most common disorders of gut–brain interaction. Adequate hydration is recommended as first-line therapy, yet evidence regarding the efficacy of sulfate- and magnesium-rich mineral water remains limited. Therefore, this study investigated its efficacy and safety in a nationwide decentralized randomized controlled trial.

**Methods:**

In this randomized, double-blind, controlled parallel-group trial, adults with chronic constipation according to the Rome IV criteria consumed either 1 L/day of sulfate- and magnesium-rich mineral water (HSM) or a low-mineralized control water (LSM) for 3 weeks following a one-week run-in period. Participants maintained a total daily fluid intake of at least 1,5L/day. The primary outcome was the change in weekly bowel movement frequency after 3 weeks. Additional analyses were conducted after 2 weeks of intervention. Secondary outcomes included stool consistency, responder rates, time to treatment response, constipation-related symptoms (PAC-SYM), constipation-related quality of life (PAC-QoL), gastrointestinal transit time, laxative use, and safety.

**Results:**

After 2 weeks (additional analysis), bowel movement frequency increased significantly more in the HSM group than in the control group (mean change: 1.31 ± 3.24 vs. 0.34 ± 2.49 bowel movements/week; *p* = 0.043). This effect was more pronounced in participants with moderate-to-severe constipation (*p* = 0.022). No significant between-group difference was observed at the end of week 3 (*p* = 0.144). Time to treatment response was significantly shorter with HSM water than with control water (median 9 vs. 16 days; *p* = 0.042), accompanied by a higher responder rate in participants with moderate-to-severe constipation (*p* = 0.043). No significant between-group differences were observed for stool consistency, constipation symptoms, quality of life, or gastrointestinal transit time. Both interventions were well tolerated, and only mild transient adverse events were reported.

**Discussion:**

Daily consumption of 1 L of sulfate- and magnesium-rich mineral water, combined with adequate hydration, improved bowel movement frequency at week two and accelerated treatment response in adults with chronic constipation, whereas the between-group difference at the registered primary endpoint (week three) was not statistically significant. These findings suggest that sulfate- and magnesium-rich mineral water may serve as a safe, non-pharmacological dietary approach for the management of chronic constipation under real-world conditions.

**Clinical trial registration:**

https://clinicaltrials.gov/, DRKS00034871.

## 1 Introduction

Chronic constipation is a common disorder of gut-brain interaction that substantially impairs quality of life and contributes to significant healthcare costs worldwide (1–3). It is mainly characterized by persistently difficult, infrequent, and/or incomplete defecation (4, 5). From a clinical perspective, a distinction is made between functional constipation (FC), irritable bowel syndrome with constipation (IBS-C), opioid-induced constipation (OIC), and functional defecation disorders (5, 6). In the context of FC, the primary focus lies on the symptoms associated with the condition itself. In the context of IBS-C, however, these symptoms are accompanied by severe episodes of abdominal pain, which is often associated with bowel movements (6). In functional disorders, the pathophysiology of constipation is multifaceted and encompasses factors such as visceral hypersensitivity, abnormalities in sensory/motor function, delayed colonic transit, and altered central perception (5). Conversely, opioid-induced constipation is characterized by diminished intestinal secretion and motility, consequent to the activation of μ-opioid receptors within the enteric nervous system (7). The term “chronic constipation” thus encompasses several conditions, yet it is important to note that it may involve different diagnostic criteria.

The most widely applied diagnostic guideline for chronic constipation are the Rome Criteria (6), which have been in operation in their 5th edition since June 2026. According to the diagnostic criteria valid from 2016 until June 2026 (Rome IV), functional constipation is classified as a disorder of gut–brain interaction and requires the presence of characteristic symptoms for at least 3 months, with symptom onset at least 6 months before diagnosis (Table 1).

**Table 1:** Diagnosis of functional constipation (FC) according to the Rome IV Questionnaire

**Symptom onset at least 6 months prior to diagnosis**			
**Nr.**	**Symptom**		**Frequency**
**1**	At least two of the following symptoms:		
	a)	Frequency: <3 bowel movements/week	
	b)	Consistency: Hard or lumpy stools (Bristol stool scale: types I and II)	Each in > 25% of bowel movements
	c)	Straining during defecation	
	d)	Sensation of incomplete evacuation	
	e)	Sensation of anorectal blockage/obstruction	
	f)	Utilization of hands to facilitate bowel movements	
**2**	Loose stools are rarely present without laxatives (Bristol stool scale: types VI and VII)		
**3**	Insufficient criteria for Irritable Bowel Syndrome (IBS)		

The heterogeneous symptom profile and differing diagnostic approaches contribute to considerable variations in reported prevalence rates. Thus, prevalence ranges from 0.7% to 81% (8), with a global average of between 12% and 15% (9, 10). It has been estimated that women are affected to a greater extent than men, with women experiencing the condition twice as often as men. Furthermore, an increase in prevalence with age has been observed (11).

Current management strategies for chronic constipation focus on a stepwise approach, initially starting with modifications of daily routines. First-line treatment is typically non-pharmacological in nature and consists of lifestyle and dietary modifications (12). Dietary fiber supplementation, notably through augmented intake of soluble fibers (e.g., psyllium), constitutes a fundamental element of therapeutic intervention (13). Concurrently, adequate hydration (1,500–2,000 mL/day) is generally recommended to ensure physiological stool consistency and bowel movements (5). In this context, mineral water has gained increasing scientific interest as a potential supportive strategy. In particular, sulfate- and magnesium-rich mineral waters may positively affect bowel function and represent a simple, non-pharmacological option in the management of chronic constipation (14). However, evidence of effectiveness on this type of mineral water is still rare (1, 15–17).

The present randomized, double-blind intervention study was conducted as a large, nationwide trial across Germany, aiming to evaluate the efficacy and safety of drinking 1 liter of sulfate- and magnesium-rich mineral water daily for 3 weeks in adults suffering from chronic constipation. Importantly, the study was designed as a fully decentralized, home-based trial, thereby capturing outcomes under real-life conditions. By relying exclusively on participant-reported assessments in a real-life setting, the study aimed to generate robust real-world evidence and provide insights into the potential benefits of mineral water consumption in this population.

## 2 Materials and methods

### 2.1 Study design

This parallel-group randomized double-blind controlled intervention study was designed to compare the effects of a sulfate- and magnesium-rich mineral water with a low mineralized mineral water on individuals suffering from chronic constipation. The trial was conducted in accordance with the CONSORT guidelines. It was designed in line with the principles of Good Clinical Practice (GCP) and the ethical standards set out in the Declaration of Helsinki. Ethical approval was authorized by the Ethics Committee of the Medical Chamber of Lower Saxony (Hannover, Germany). Trial registration was undertaken at the German Clinical Trials Register (DRKS) (DRKS00034871) prior to the start of recruitment.

### 2.2 Participants and procedure

A total of 157 healthy individuals were recruited from the general population between August 2024 and January 2026. The Germany-wide recruitment was realized online via various advertisements, mainly in social media. Interested individuals were directed to an online screening questionnaire to assess eligibility. This questionnaire consisted of the Rome IV Questionnaire (18) and a short survey to gather general health information. Individuals who were eligible were subsequently invited to register for study participation. After registration, all eligible participants were randomly assigned in a 1:1 ratio to one of the two intervention groups under double-blind conditions. Randomization was performed by an independent researcher who was not involved in recruitment, data collection, or outcome adjudication. The random allocation sequence was computer-generated using stratified block-randomization according to the covariates sex, age, and BMI. The allocation file was stored in a secure, access-restricted database accessible only to the independent researcher responsible for randomization.

Detailed information about the study objectives, procedures, potential risks and benefits were easily accessible online at the screening platform for every interested person and were send to the eligible individuals via e-mail before enrollment. Informed consent was obtained electronically from each participant before enrollment.

Eligible participants were healthy adults between the age of 18 and 75 who reported information sufficient to diagnose chronic constipation according to the Rome IV Questionnaire for adults (module: bowel disorders) (18). Individuals diagnosed as irritable bowel syndrome with predominant constipation were also invited to participate. Further inclusion criteria encompassed a total fluid consumption of at least 1,500 mL/day, a body mass index ranging from 18.5 to 35 kg/m^2^, a daily fiber intake by a consumption of vegetables, fruits and/or whole-grain products, and a normal, age-appropriate level of physical activity. Individuals with medication or supplements affecting constipation were eligible to participate as long as there had been no change in their regimen within the previous month before the individual start of the study. Health status was assessed using the screening questionnaire that excluded individuals with diagnosed chronic gastrointestinal conditions, untreated thyroid disorders, cancer within the last 2 years, liver and kidney diseases as well as pregnant or breastfeeding women. Exclusion also applied to those individuals consuming iron supplements.

The study was carried out in several waves between September 2024 and March 2026. Each wave started with a 7-day run-in-phase to ensure that participants consumed the minimal required amount of fluids. In this phase, participants were instructed to consume 1,500–2,000 mL/day of a low mineralized spring water (LM water) to be drunken throughout the day. During the following three intervention weeks, participants were requested to drink 1,000 mL/day of the respective study water. The HSM group consumed a sulfate- and magnesium-rich mineral water (HSM water), while LSM group was given a mineral water low in sulfate and magnesium (LSM water). To meet the required minimum intake of 1,500 mL of fluid per day, participants were allowed to drink an additional 500 mL of the low mineralized spring water (LM water), which had also been consumed during the run-in-week. Additional fluid intake was allowed throughout the study. However, other mineral water than the delivered mineral water was not permitted.

Participants were requested to continue their medication and supplement intake, as well as to maintain their dietary and exercise routines throughout the study.

Mineral water was delivered to the participant’s homes before the start of the run-in-phase in combination with a detailed instruction of the study procedure.

### 2.3 Test products

Mineral water used in this study can be distinguished in two categories: high mineralized mineral water (HSM water) and low mineralized water (LSM water and LM water). The high mineralized water (2,240 mg/L; St. Antonin; HSM) served as the study water and was characterized by a high sulfate (1,450 mg/L), magnesium (89 mg/L), and calcium (568 mg/L) content, according to the European Directive 2009/54/EC (19). The LSM water (267 mg/L St. Francois; LSM) was a low-mineralized mineral water, low in sulfate (27 mg/L) and magnesium (19 mg/L). It served as the control water. Both mineral waters (HSM, LSM) were consumed during the intervention period to test the study hypotheses. To ensure blinding, a second low-mineralized water (Cristaline, Ste. Cecile; LM) was used during the run-in-week and the intervention weeks to ensure the required amount of fluids. In contradistinction to the study waters, which are both mineral waters, the LM water is to be classified as spring water. The average mineral content of all waters is listed in Table 2.

**Table 2:** Mineral content of the mineral waters.

Mineral	Test products (time: intervention)		Additional water (time: run-in + intervention)
	Water 1 (HSM) (St. Antonin) (mg/L)	Water 2 (LSM) (St. François) (mg/L)	Water 3 (LM) (Cristaline, Ste. Cecile) (mg/L)
Sulfate	1,450	27	6
Magnesium	89	19	25
Calcium	568	72	39
Potassium	3	0.6	1.5
Sodium	7.9	5.5	19
Chloride	9	5	5
Bicarbonate	359	285	290
Total mineral content	2,440 (high)	267 (low)	270 (low)
Group	HSM group	LSM group	HSM + LSM group

HSM, High sulfate and magnesium; LSM, low sulfate and magnesium.

### 2.4 Outcomes

Severity of constipation (Rome Score) was calculated using data obtained from the screening questionnaire. Scoring algorithm is shown in Table 3. Participants were subsequently assigned to one of three tertile categories, based on the maximum score of 90. This categorization enabled the classification of participants as mild (points 0–29; cat. I), moderate (points 30–59; cat. II), or severe (points 60–90; cat. III) constipated.

**Table 3:** Rome Scoring (Rome IV) according to the frequency of symptoms

Variable (*)	Score value	Answers (each)	Score
Abdominal pain close to bowel movements (Q 41)	0-10	0% (never)	0
Abdominal pain and softer/harder stool (Q 42)	0-10	10%	1
Abdominal pain and more or less frequent bowel movements (Q 43)	0-10	20%	2
Hard or lumpy stools (type I and II) (Q 49)	0-10	30%	3
Frequency: <3/week (Q 51)	0-10	40%	4
Straining during defecation (Q 52)	0-10	50%	5
Sensation of incomplete evacuation (Q 53)	0-10	60%	6
Sensation of anorectal obstruction/blockage (Q 54)	0-10	70%	7
Manual maneuvers to facilitate defecation (Q 55)	0-10	80%	8
		90%	9
**Maximum total score**	**90**	100% (always)	10

^*^Question number of the Rome IV questionnaire.

To assess the primary and secondary outcomes described below, participants completed online questionnaires at the end of the run-in-week (baseline) and at the end of the intervention period. Furthermore, participants were required to fill in stool protocols (quantifying amount and type of bowel movements) and fluid protocols on a daily basis throughout the study period. Baseline data were assessed after the run-in-week and intervention data were evaluated after intervention week two and three. All data were self-reported data. In the event of any ambiguity, the participants were contacted immediately to resolve the issue.

*Primary parameter* was defined as the change in mean number of bowel movements per week between baseline and intervention week three (defecation frequency). Additional analyses were conducted between baseline and intervention week two. A daily protocol was used to facilitate the assessment, which was recorded as part of the self-evaluation diary. The participants were required to upload the requested data to an online tool or send it via messenger/e-mail to the study team at the end of each study week.

*Secondary parameters* were defined as the average stool type during each week (stool consistency), responder rates at the end of intervention week two and three (frequency, consistency), time to response (frequency, consistency), severity of constipation symptoms [PAC-SYM^©^ (20)], constipation-related quality of life [PAC-QoL ^©^ (21)], transit time, safety (adverse events) and the use of laxatives.

Stool consistency was assessed using a daily protocol as part of the self-evaluation diary. In this protocol, participants reported the stool type for each bowel movement according to the Bristol stool scale. Mean stool type was calculated as a weekly average for each participant. On days with more than one bowel movement, each individual stool type was taken into account.

Participants were defined as responders based on the components of the Rome IV criteria:

1. Responder (frequency): (a) increase of ≥ 2 bowel movements/week as compared to baseline or (b) ≥ 3 bowel movements/week, if baseline ≤2.
2. Responder (consistency): <25% of bowel movements with type 1 + 2 of Bristol stool scale, if baseline ≥ 25%.

Time to treatment response was analyzed using Kaplan–Meier curves. For bowel movement frequency, baseline was defined as the individual mean number of bowel movements per week during the run-in period. During the intervention, bowel movement frequency and stool consistency were evaluated using 7-day rolling averages and compared with the respective baseline values. During the first 7 days of the intervention, the rolling average was calculated cumulatively using all available intervention days (day 1; days 1–2; days 1–3; …; days 1–7). From day 7 onward, the rolling average was calculated using a moving 7-day window including the respective day and the preceding 6 days. The first day of treatment response was defined as the first intervention day on which the participant fulfilled the respective responder criterion.

Symptoms and quality of life were evaluated using validated questionnaires for constipation (PAC-SYM^©^, PAC-QoL ^©^). Questionnaires were filled in after the run-in-week and at the end of the intervention.

Gastrointestinal transit time was assessed during the run-in period and during the final intervention week using cookies colored with blue food dye. Participants were instructed to consume the cookies together with a meal at the midpoint of each assessment period to increase the volume of the colored bolus. Transit time was calculated as the interval between cookie consumption and the first appearance of blue-green colored feces. In order to prevent any unintended discoloration of the stool, participants were instructed to refrain from the consumption of red beets or spinach during these two assessment periods.

Safety of mineral water consumption was monitored using weekly protocols as part of the self-evaluation diary. All documented adverse events (AE) were subsequently categorized as possibly related or unrelated to the intervention. At the end of the intervention, participants were interrogated about their subjective perceptions regarding the observed reduction in laxative use. These data were utilized to assess laxative use.

Compliance was verified using daily fluid logs. Participants were requested to provide comprehensive data on their total fluid intake, using a daily protocol consisting of multiple fluid categories (coffee/tea, tap water, juices, soft drinks, alcoholic beverages, additional beverages, soup) and water intake (mineral water, spring water). To monitor water consumption, participants were asked to document the amount of test water and LM water in a daily protocol. Participants were considered to be compliant if they consumed a minimum of 1,500 mL of fluids (mineral water, spring water, other beverages) per day (average per study week). Moreover, consumption of less than 700 mL of the study water (HSB, LSB) per day resulted in exclusion from the analysis. In the event of fluid intake being maintained at ± 1,750 mL/week (250 mL/day), as compared to the run-in week, the total fluid intake was deemed to be stable. A decrease in fluid intake of more than 1,750 mL/week (250 mL/d) was considered to be a decrease. An increase in fluid intake of more than 1750 mL/week (250 mL/d) compared to the baseline was considered to be an increase in fluid consumption.

Fiber intake and physical activity were monitored to evaluate the maintenance of optimal study conditions.

### 2.5 Sample size calculation

*A priori* power analysis was performed to estimate the required sample size. Using the frequency of bowel movements as the outcome measure, an effect size of Cohen’s *d* = 0.58 was derived from a previous study examining the effect of sulfate- and magnesium-rich mineral water on functional constipation in middle-aged individuals (15). The original sample-size calculation was based on a conservative target power of 95% to reduce the probability of type II error. Under this assumption, the target sample size was 206 participants, including an anticipated dropout rate of 20%. Ultimately, 157 participants were randomized, corresponding to 76% of the registered target sample size. Although the original target based on 95% power was not reached, a conventional calculation using 80% power and a two-sided type I error rate of 5% indicated that 126 participants, including the anticipated 20% dropout rate, were required. The achieved sample size therefore exceeded the number required under the conventional 80% power criterion. Accordingly, the shortfall relative to the conservatively defined 95%-power target is not expected to have materially compromised the study’s ability to detect the prespecified effect.

### 2.6 Statistical analysis

Distribution of all data was assessed using Shapiro–Wilk test and Q-Q-plots. Non-normally distributed data were transformed using the logarithm (log) transformation or square-root (sqrt) transformation to improve normal distribution. Descriptive statistics are presented on the original scale for ease of interpretation. Notwithstanding the partial absence of normal distribution, analyses of variance (ANOVAs) were conducted, as variance analyses are regarded as being robust to violations of the normal distribution assumption when a sufficiently large sample size is employed (central limit theorem, *n* > 50) (22). Data are presented as mean ± standard deviation or median and interquartile range (IQR), depending on the distribution.

Differences in baseline characteristics between the two intervention groups were analyzed using the independent-sample *t*-test for continuous variables. Categorical variables were compared using Chi-squared test. To assess differences between the intervention groups, univariate ANOVAs were employed to analyze changes from baseline to the end of week two (frequency, consistency) and three (frequency, consistency, fiber intake, PAC-SYM^©^, PAC-QoL ^©^, transit time). In a further step, outcomes were controlled by age and sex using ANCOVAS (frequency, consistency). The influence of the changes in total fluid consumption on the frequency of bowel movements was examined using a two-way ANOVA with interaction. Time to treatment response was illustrated using Kaplan–Meier curves and differences were calculated using the log-rank test. In addition, repeated-measures analyses of variance (rmANOVA) were performed between baseline and the end of the intervention to compare inner-group differences for fiber intake and physical activity. Changes in total fluid intake over time were analyzed computing a two-way repeated measures ANOVA. Sensitivity analyses were conducted to examine the effects on individuals with moderate and severe constipation, excluding subjects from the lowest tertile of the Rome score. It was deemed inappropriate to conduct single analyses exclusively for mildly constipated participants, given the limited number of individuals within this specific subgroup.

The primary analyses were conducted using a per-protocol (PP) approach based on available data. Participants were included in the respective analyses if outcome data were available for the relevant study time point and no major protocol deviations affecting the assessment of the intervention had occurred. Accordingly, the number of participants included in the analyses differed across time points. Missing data were not imputed. To assess the robustness of the findings, intention-to-treat (ITT) analyses are additionally provided in the supplementary material.

All statistical analyses were performed using SPSS Software for Windows (Version 30.0.0.0; SPSS Inc., Chicago, IL, United states). Two-sided *p* < 0.05 were considered as statistically significant.

## 3 Results

### 3.1 Study population

A total of 473 individuals completed the online screening questionnaire, of whom 157 (144 women, 13 men) were randomized (Figure 1). A total of 75 participants were assigned to the control group and 82 to the intervention group. Of these, 7 individuals did not take part in the baseline survey. Therefore, baseline data from 150 participants (HSM group: *n* = 80, LSM group: *n* = 70) were available. No participant was excluded due to insufficient water consumption. Two individuals did not provide stool data at baseline. Therefore, they were excluded from the ITT analysis of stool-related outcomes (*N* = 148). Moreover, stool consistency data were unavailable for one participant at baseline, resulting in an ITT analysis of stool consistency based on 147 participants.

**Figure 1:**
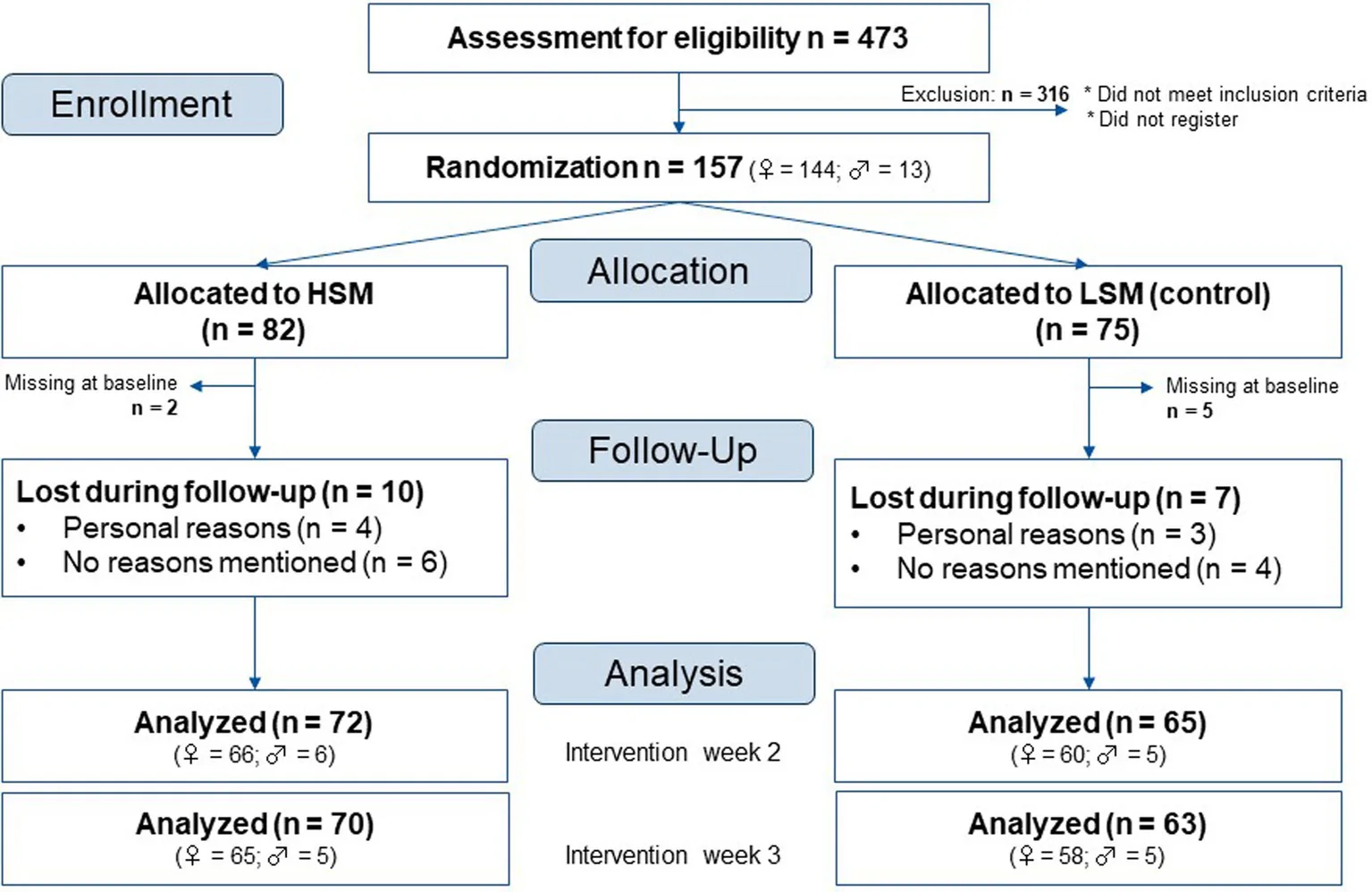
CONSORT flow diagram. HSM = High sulfate and magnesium; LSM = low sulfate and magnesium (PP analysis).

Until the end of intervention week two 17 individuals were lost. Of these, seven mentioned personal reasons, while the others did not communicate any reason. Consequently, the effect of the two-week consumption of mineral water was analyzed in the PP-approach using data from 137 individuals. Covering the whole three-week intervention period, data from 133 individuals were available.

General baseline characteristics of both intervention groups are presented in Table 4. The study cohort was predominantly female (91.3%), moderately (47.3%) or severely (43.3%) constipated, and characterized by normal weight with a mean BMI of 25.7 ± 4.2 kg/m^2^. No differences in sex distribution, anthropometric variables, or Rome Score existed before the start of the intervention (*p* > 0.05).

**Table 4:** Baseline characteristics of the study cohort.

Variables/parameters	Total *N* = 150 (100%)	HSM *n* = 80 (%)	LSM *n* = 70 (%)	*p*-value
Gender				
Male [*n* (%)]	13 (8.7%)	8 (10%)	8 (7.1%)	0.535^a^
Female [*n* (%)]	137 (91.3%)	72 (90%)	65 (92.9%)	
Anthropometry*				
Age [years]	49.2 ± 12.0	49.1 ± 12.4	49.3 ± 11.6	0.952^b^
BMI [kg/m^2^]	25.7 ± 4.2	25.9 ± 4.5	25.6 ± 4.0	0.707^b^
Severity of constipation (Rome score)				
Mild [n (%)]	14 (9.3%)	8 (10%)	6 (8.6%)	0.677^a^
Moderate [n (%)]	71 (47.3%)	40 (50%)	31 (44.3%)	
Severe [n (%)]	65 (43.3%)	32 (40%)	33 (47.1%)	

*Data are shown as mean ± SD. BMI, body mass index, HSM, High sulfate and magnesium; LSM, low sulfate and magnesium; SD, standard deviation. ^a^Chi-squared-test, ^b^*T*-test.

### 3.2 Physical activity, fiber intake, and fluid consumption

Participants maintained their physical activity levels during the intervention period (p_HSM_ = 0.792; p_LSM_ = 0.783). Fiber intake decreased significantly in both intervention groups (both *p* < 0.001), but not between groups (change *p* = 0,317). In terms of total fluid intake, no differences were observed over the course of time between the two intervention groups (*p* = 0.537). 64% (*n* = 87) of the participants showed a stable consumption over the course of the intervention. On the contrary, 13.2% (*n* = 18) of the study cohort showed an increase in fluid consumption, while it decreased in 22.8% (*n* = 31) of the participants.

### 3.3 Primary outcome

Following a period of three intervention weeks, no significant between-group differences were observed in the analysis based on log10-transformed data (*p* = 0.144) (Table 5). An additional analysis conducted with the data from week two showed that bowel movement frequency increased in both groups, but the increase over time reached statistical significance only in the HSM group (p_HSM_ < 0.001; p_LSM_ = 0.401). Accordingly, the change from baseline to week two was significantly greater in the HSM group than in the LSM group (*p* = 0.043) (Table 6). Adjustment for age and gender yielded similar results. In a subgroup analysis including participants in the second and third tertiles of the Rome score, this association was more pronounced than in the total study group (Supplementary Table S1) (*p* = 0.022). These findings suggest a possible short-term effect at week two.

**Table 5:** Changes in frequency and consistency from baseline to intervention week three.

Parameter	HSM	LSM	Estimated treatment effect (HSM vs. LSM) (95% CI)	*p*-value^a^
Frequency*	1.19 ± 2.31	0.62 ± 3.26	0.017 (−0.006 to 0.040)	0.144
Consistency**	0.47 ± 1.42	0.83 ± 1.49	−0.366 (−0.866 to 0.134)	0.150

Data are presented as mean change ± SD on the original scale. Between-group effect estimates, 95% confidence intervals, and *p*-values are based on analyses of log-transformed data (frequency) or untransformed data (consistency). Positive effect estimates indicate higher values in the HSM group than in the LSM group. HSM, High sulfate and magnesium; LSM, low sulfate and magnesium; SD, standard deviation. ^a^One-way ANOVA. *n_HSM_ = 70; n_LSM_ = 63. **n_HSM_ = 70; n_LSM_ = 62.

**Table 6:** Changes in frequency and consistency from baseline to intervention week two.

Parameter	HSM	LSM	Estimated treatment effect (HSM vs. LSM) (95% CI)	*p*-value^a^
Frequency*	1.31 ± 3.24	0.34 ± 2.49	0.026 (0.001 to 0.050)	0.043
Consistency**	0.45 ± 1.52	0.47 ± 1.31	−0.014 (−0.499 to 0.471)	0.955

Data are presented as mean change ± SD on the original scale. Between-group effect estimates, 95% confidence intervals, and *p*-values are based on analyses of log-transformed data (frequency) or untransformed data (consistency). Positive effect estimates indicate higher values in the HSM group than in the LSM group. HSM, High sulfate and magnesium; LSM, low sulfate and magnesium; SD, standard deviation. ^a^One-way ANOVA. *n_HSM_ = 72; n_LSM_ = 65. **n_HSM_ = 72; n_LSM_ = 64.

Supplementary intention-to-treat analyses yielded comparable results (Supplementary Tables S2–S4).

The alterations in physiological and pathophysiological bowel movement metrics are shown in Figures 2, 3. With regard to the pathophysiological numbers of bowel movements (0–2/week), a decrease was observed only in the HSM group, which was accompanied by an increase in the physiological numbers (≥3). Conversely, the LSM group showed no or only marginal variations within each category between baseline and intervention week two or three.

**Figure 2:**
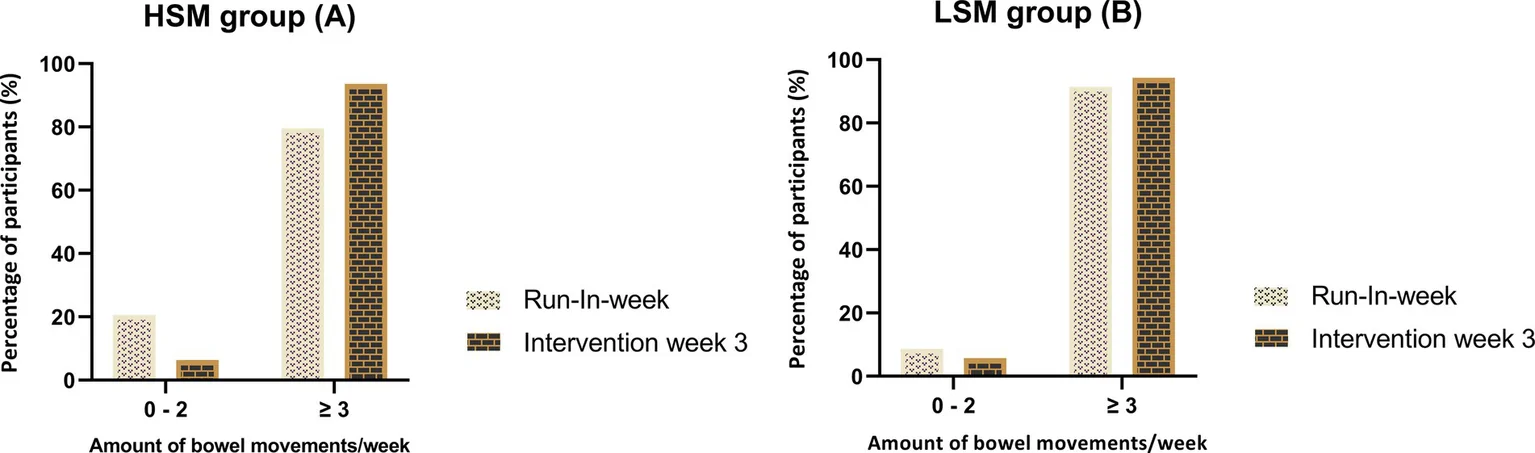
Frequency of bowel movements depending on the amount of bowel movements/week. Changes are shown for the HSM group **(A)** and the LSM group **(B)** from baseline to intervention week three. HSM = High sulfate and magnesium; LSM = low sulfate and magnesium.

**Figure 3:**
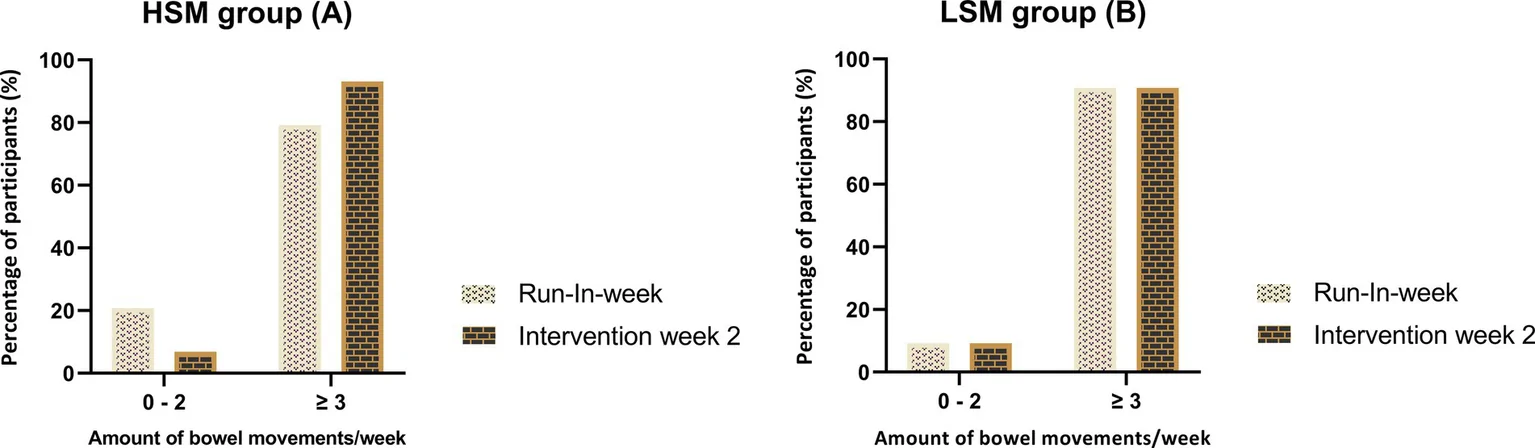
Frequency of bowel movements depending on the amount of bowel movements/week. Changes are shown for the HSM group **(A)** and the LSM group **(B)** from baseline to intervention week two. HSM = High sulfate and magnesium; LSM = low sulfate and magnesium.

Examining the influence of changes in total fluid consumption on frequency, revealed no significant interaction covering the whole intervention period (*p* = 0.629). However, a significant interaction between group and fluid intake occurred after two weeks of mineral water consumption (*p* = 0.038). Further analysis indicated that significant group differences existed among individuals who increased their total fluid intake by at least 1,750 mL/week (250 mL/d) compared to the run-in-week (*p* = 0.001). Differences between the groups are shown in Figure 4.

**Figure 4:**
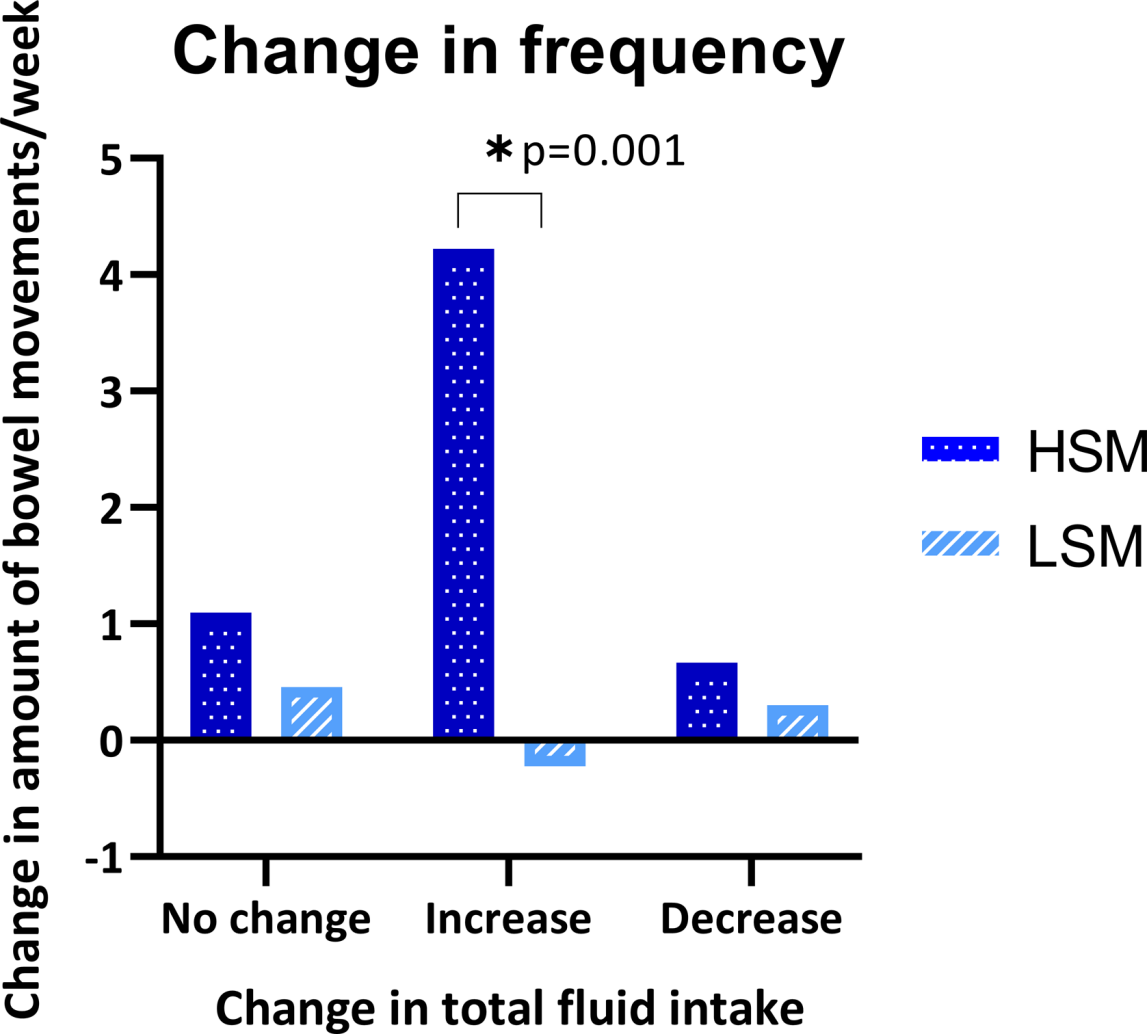
Changes in frequency of bowel movements depending on the change in total fluid consumption. Changes are shown for both intervention groups from baseline to intervention week two. HSM = High sulfate and magnesium; LSM = low sulfate and magnesium.

### 3.4 Secondary outcomes

#### 3.4.1 Time to treatment response (frequency)

The Kaplan–Meier curve for the treatment response in frequency is shown in Figure 5. Time to treatment response was shorter in the HSM group compared to the LSM group (median: 9 days vs. 16 days). Differences between the study groups were significant (responders *n* = 52 vs. *n* = 37; *p* = 0.042). Taking into account only individuals characterized by a moderate to severe constipation (categories II + III of the Rome Score), associations even strengthened, showing significant differences between the study groups with an even shorter time to response in the HSM group (responders: *n* = 49 vs. *n* = 34; *p* = 0.016; median: 8 vs. 16 days).

**Figure 5:**
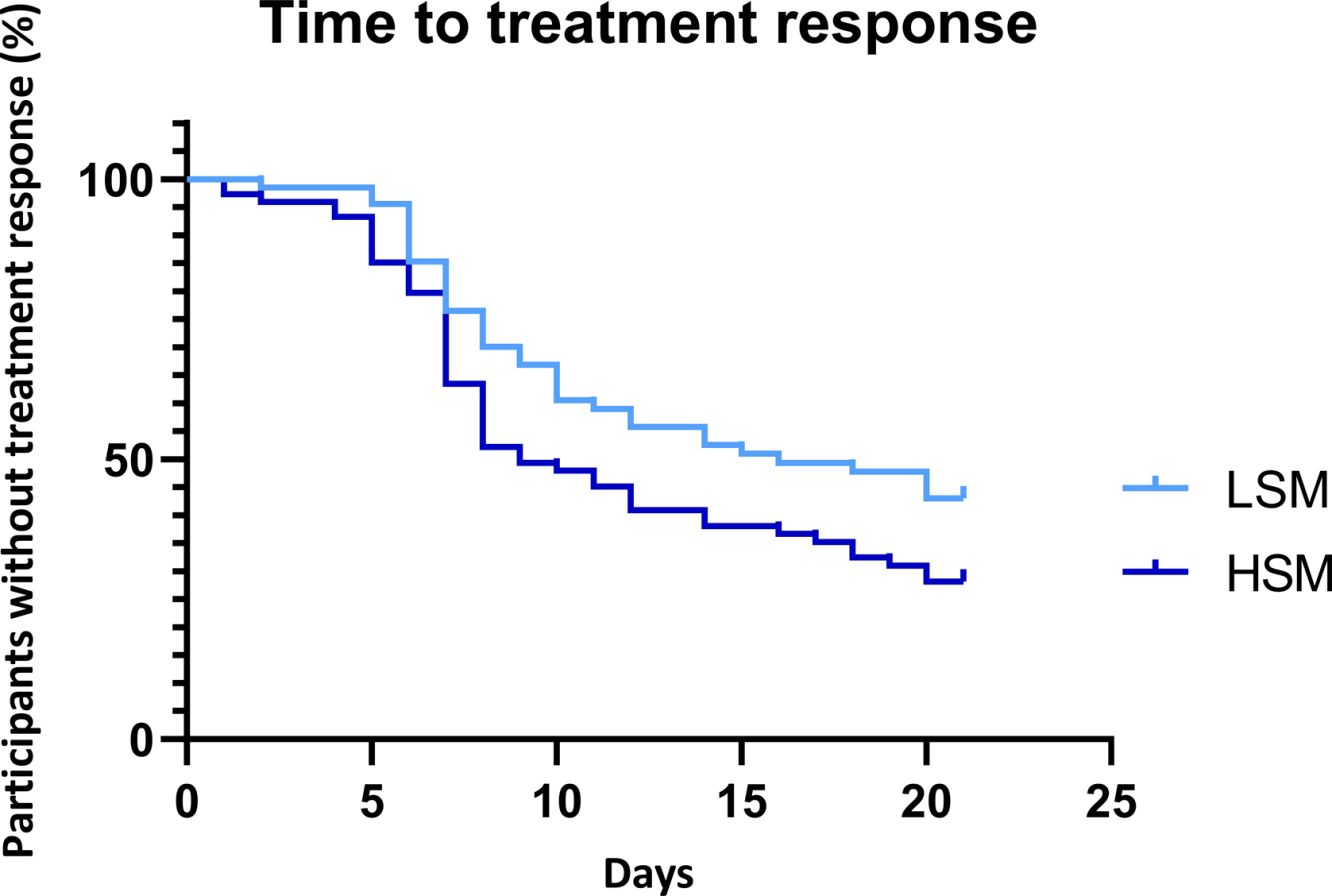
Kaplan-Meier curves showing time to treatment response in bowel movement frequency from baseline to the end of the intervention, displayed as the probability of remaining without treatment response over time. HSM = High sulfate and magnesium; LSM = low sulfate and magnesium.

#### 3.4.2 Responder rate (frequency)

In accordance with the statistical analysis of the time to treatment response in frequency, the responder analysis indicates that the HSM group exhibited a higher responder rate in comparison to the LSM group after two (45.8% vs. 30.8%) and three weeks of intervention (54.3% vs. 33.3%). While the difference after two weeks of intervention did not reach statistical significance (*p* = 0.081), a significant difference occurred at the end of the intervention week three (*p* = 0.023). Again, associations were strengthened in the subgroup analysis comprising individuals diagnosed with moderate or severe constipation after two (*p* = 0.043) and three weeks of intervention (*p* = 0.010).

#### 3.4.3 Laxative use and safety

In both intervention groups, the majority of the participants reported no perceived change in laxative use (HSM = 88.9%; LSM = 93.5%) from the beginning to the end of the intervention. However, among those who reported a perceived reduction in laxative use, the percentage was higher in the HSM group than in the LSM group (11.1% vs. 6.5%).

Safety was assessed on data from the entire study group (*N* = 150), encompassing 3 weeks of intervention for each participant. A total of 16 different adverse events (HSM = 10; LSM = 9) were reported by 20 individuals (HSM = 10; LSM = 10) during the whole intervention period. About half of the AEs were considered to be independent to the intervention. On the contrary, some minor adverse events could have possibly occurred as a consequence of the intervention. However, these symptoms were usually temporary.

#### 3.4.4 Others

Regarding the other secondary outcomes (changes in consistency, symptoms, quality of life, transit time) no statistically significant differences were identified between the intervention groups (all *p* > 0.05), neither after two weeks nor after three weeks of intervention. Nevertheless, improvements were observed in all parameters over time in both intervention groups.

## 4 Discussion

### 4.1 Outcomes

This randomized, double-blind, controlled intervention study, conducted with individuals suffering from chronic constipation, indicates that the consumption of mineral water rich in sulfate and magnesium can improve bowel function. Between-group differences were most evident for bowel movement frequency after 2 weeks of intervention.

However, significant improvements in constipation symptoms were only observed after a period of 2 weeks. As the intervention progressed, the study groups demonstrated a consistent convergence, with no significant differences detected by the end of the study. This finding aligns with the results of the majority of the former studies that evaluated data at different time points (1, 15). For example, the research group headed by Dupont et al. (1) demonstrated significant increases in bowel movement frequency after a period of 2 weeks. By the end of the study (week 4), however, no significant differences between the groups could be identified. A second study by the same research group examined the effects of consuming sulfate- and magnesium-rich mineral water for 2 weeks, demonstrating improvements in frequency and consistency (17). In this study, the effects of a longer intake were not tested. Furthermore, Naumann et al. (15) confirmed improvements in bowel movement frequency after a period of 3 weeks, but not after 6 weeks. In contrast, only a single study has demonstrated beneficial effects following a prolonged consumption period of 6 weeks (16). In this study, both the frequency and consistency of stools changed significantly different between the intervention group, which consumed sulfate- and magnesium-rich mineral water, and the control group. It should be noted, however, that this mineral water had a very high magnesium content (1,000 mg/L) and the amount consumed exceeded the upper limit of safe intake for magnesium (250 mg/d) in water (23). Consequently, the observed effects are presumably attributable, at least in part, to the potent laxative effect of the elevated magnesium doses.

In the present intervention study, the change in the number of bowel movements was selected as the primary outcome, as it represents the most objectifiable parameter of symptoms associated with constipation. From the run-in week to the end of the intervention week 2, the number of stools per week increased by 1.31 ± 3.24 in the HSM group, while there was no significant increase in the control group. This is similar to the findings from previous studies, where an increase between 2 and 3 stools/week was demonstrated (15, 16). Hence, changes in the present study are at the lower end of the span. It is noteworthy, however, that the participants in this study met the criteria for chronic obstipation. Nevertheless, the vast majority of them already showed physiological numbers (≥ 3/week) regarding stool frequency at baseline, and there was no upper limit for bowel movements at the time of inclusion. By contrast, the aforementioned studies included only those individuals who reported bowel movements between 2 and 4 per week. Consequently, these studies selected for a specific symptom of obstipation. In the present study, no such preselection was employed. All participants who met the Rome criteria for chronic constipation were included, regardless of the presence of single symptoms. This might be the reason for the discrepancies between the findings.

Insufficient fluid intake is widely recognized as a significant contributing factor to chronic constipation (10, 24–27). The hypothesis, stated by Naumann et al. (15), which suggests that an increase in fluid intake may be responsible for the observed improvements even in the control group, is therefore considered to be plausible. This association was shown in an earlier study evaluating fluid intake in combination with an elevated fiber intake (28). As both groups consumed an equivalent amount of dietary fiber, an increase in bowel movements was observed in both groups. However, participants who consumed 2,100 mL water per day showed a higher stool frequency than participants in the control group consuming only 1,100 mL/day (ad libitum). Therefore, the increase in fluid intake is to be considered as a driving component. On the other hand, it should be noted that increasing fluid intake beyond the recommended amount of 1,500–2,000 mL/day is not regarded to be associated with a further improvement in constipation symptoms (12). The present study provides further evidence to support these relationships. During the run-in phase, all participants were instructed to consume at least 1,500 mL of fluids per day, to ensure balanced hydration before intervention. This minimum intake was also achieved during the intervention period. Although fluid intake remained largely stable in most participants, some showed an increase or decrease over time. In the subgroup of participants with increased fluid intake during the intervention, significant differences in bowel movement frequency were observed between the intervention groups. Because this subgroup classification was based on a post-randomization behavioral change rather than a randomized factor, this finding should be considered exploratory. Nevertheless, this finding suggests that the observed group difference may not be explained by fluid volume alone and is compatible with a possible role of mineral water composition. This interpretation requires confirmation in studies specifically designed to disentangle the effects of fluid volume and water composition.

In addition to the effect on the number of bowel movements per week, the time to onset of the effect may also be a relevant consideration for individuals affected by constipation. For this reason, the time to treatment response was calculated using Kaplan–Meier analysis. The analysis revealed that the effect occurred in a significantly shorter time span in the HSM group as compared to the control group (9 days vs. 16 days), indicating a superiority of sulfate- and magnesium-rich mineral water over a low-mineralized mineral water. Similar results are reported by a single previous study (17). In its 2-weeks lasting intervention, treatment response occurred after 6.4 ± 0.6 days of sulfate-rich mineral water consumption.

The evaluation of the response rate provides additional evidence that supports the same conclusion. The initial analysis of the entire cohort yielded only preliminary indications of potential group differences. Nevertheless, when solely those individuals afflicted with moderate to severe constipation were under consideration, significant disparities became evident between the two groups: individuals who drank the sulfate-rich mineral water exhibited a significant higher response rate than those who consumed the control water. A comparison between the response rates documented in earlier studies and those observed in the present study is not possible. The analysis of responders has been exclusively undertaken by Dupont’s research group, with no other research groups having published such an analysis to date (1, 17). However, the basis for calculation differs, as Dupont’s analysis took into account both frequency and consistency in one analysis. It is acknowledged that not all individuals experiencing constipation necessarily meet both criteria. Therefore, the two parameters were decoupled in the present study.

The evaluation of data regarding stool consistency demonstrated a significant improvement over time. Nevertheless, no significant differences were identified between the two study groups in this particular domain. The same applies to the evaluation of the constipation-related symptom severity and quality of life. It is imperative to acknowledge that both the assessment of stool consistency and the evaluation of symptom severity and quality of life are highly subjective parameters, which are influenced by numerous factors. This can lead to bias. For instance, Dupont et al. demonstrated that, while there was a relatively high degree of agreement between expert opinion and participant assessment regarding stool consistency, this agreement was only 79% (17). Consequently, a considerable amount of variance remains, which can only be reduced through a substantial study effort. In the context of a study group comprising fewer than 200 participants, as it was the case in this study, such a variation could potentially be decisive for the outcomes, unless it is distributed uniformly across both study groups. Consequently, the finding of non-significant differences between groups is not unexpected. On the contrary, previous studies showed significant differences at least at some examination points (1, 15, 16). However, it is worth noting that the changes in stool consistency, examined as changes in Bristol stool scale varying from types I to VII, were rather minor. For instance, Naumann et al. (15) reported a change of approximately 1.0 point, while Bothe (16) reported a change of approximately 1.3 on the Bristol stool scale. The remaining publications only reported the number of different stool types classified as pathophysiological (types I–II and VI–VII) or physiological (types III–IV) (1, 17). No changes in mean consistency were reported.

Combining the results of former studies, a recent meta-analysis was able to show a significant higher responder rate after the consumption of highly mineralized mineral water compared to low-mineralized mineral water (29). Including the present findings might strengthen this association. By contrast, this meta-analysis did not find a significant difference in stool frequency between both types of mineral water. However, a slight tendency toward significance was reported (Mean difference + 0.41; *p* = 0.08). Adding the findings from the present study may change the overall effect. Regarding stool consistency no meta-analysis of the data could be done due to different reporting methods. Consequently, it is not feasible to estimate the combined effect on stool consistency.

There is a general consensus that a sufficient intake of dietary fiber, combined with an adequate fluid intake, beneficially effects bowel function (6, 10). For this reason, dietary fiber intake was monitored at the beginning and end of the study, to evaluate possible changes over the course of the intervention. Although the participants had been instructed to maintain a consistent diet, analysis of the data revealed a significant decrease in fiber intake in both intervention groups. It can only be speculated about the underlying causes, but the improvement in stool consistency and frequency observed over time in both groups may have been a reason for the reduction in fiber intake. However, a bias in the study results due to this reduction is unlikely, as a reduction in fiber intake was observed to a similar extent in both groups. Furthermore, an improvement in stool frequency was observed, which occurred despite a reduction in fiber intake. This suggests that the increase in stool frequency may be attributable to the intervention and is unlikely to be explained by a decrease in dietary fiber intake. However, this observation should be interpreted cautiously and not as independent evidence of intervention effectiveness.

The present findings indicate that participants reported a perceived reduction in laxative use over the course of the study. This finding provides valuable insight into participants’ treatment experience and could indicate improved patient satisfaction or a subjective sense of symptom relief, both of which represent meaningful outcomes in the context of gastrointestinal interventions.

Safety profile of the sulfate- and magnesium-rich mineral water was very good in this intervention study. Only one participant showed a tendency to a pathophysiological stool type (Bristol stool scale types 5 and 6) after the consumption of 1 L of HSM water. However, the mean stool type reported by this participant had already attained a value of 3.2 on the Bristol stool scale during the run-in period. The findings of this study are consistent with those of earlier research on sulfate-rich mineral water, which has demonstrated an improvement in stool consistency without causing diarrhea (1, 15, 16). Further common complaints like nausea, bloating, stomach pain, intestinal cramps and feeling of fullness are rather unlikely to be caused by the mineral water consumption itself as it occurred in both intervention groups with the same frequency (n_HSM_ = 36, n_LSM_ = 36). However, these are gastrointestinal symptoms which are commonly associated with constipation (30). Therefore, they may also be indicative for the underlying disease and were not classified as adverse events. In fact, some individuals who reported these symptoms as adverse events claimed to have experienced them prior to the start of the study.

### 4.2 Mechanisms

The laxative effect demonstrated in the present study occurred due to the combined action of two components of HSM water: sulfate and magnesium (Figure 6). These minerals are both known to cause an osmotic reaction in the gastrointestinal tract due to the slow und partially incomplete absorption of the ions (14). Sulfate-rich waters, particularly those containing magnesium sulfate and sodium sulfate, create a hypertonic environment in the intestinal lumen, leading to rapid gastric transit and the retention of water within the gut (31). This osmotic water influx increases fecal volume, reduces stool consistency, and stimulates intestinal motility, thereby facilitating defecation (31). The limited absorption of sulfate in the intestine and the dose-dependent, partial absorption of magnesium (typically 30–50%) contribute to this luminal osmotic effect (14). In addition, magnesium sulfate appears to act through several complementary mechanisms beyond osmotic activity. Experimental studies have shown that magnesium sulfate can increase the expression of aquaporin-3 (AQP3), a key regulator of colonic water transport, and modulate osmotic pressure within the colon (32). Other proposed pathways include the stimulation of cholecystokinin (CCK), peptide YY (PYY), and nitric oxide synthase (NOS), which may enhance intestinal motility and relax circular smooth muscle cells (14, 33–35). These processes are facilitated by transcellular magnesium transport via TRPM6 and TRPM7 channels (14). While osmotic action remains the primary mechanism for sulfates, magnesium contributes to additional regulatory effects on gut motility and epithelial transport (14). Together, these mechanisms explain the observed increase in stool frequency associated with the consumption of magnesium- and sulfate-rich mineral waters.

**Figure 6:**
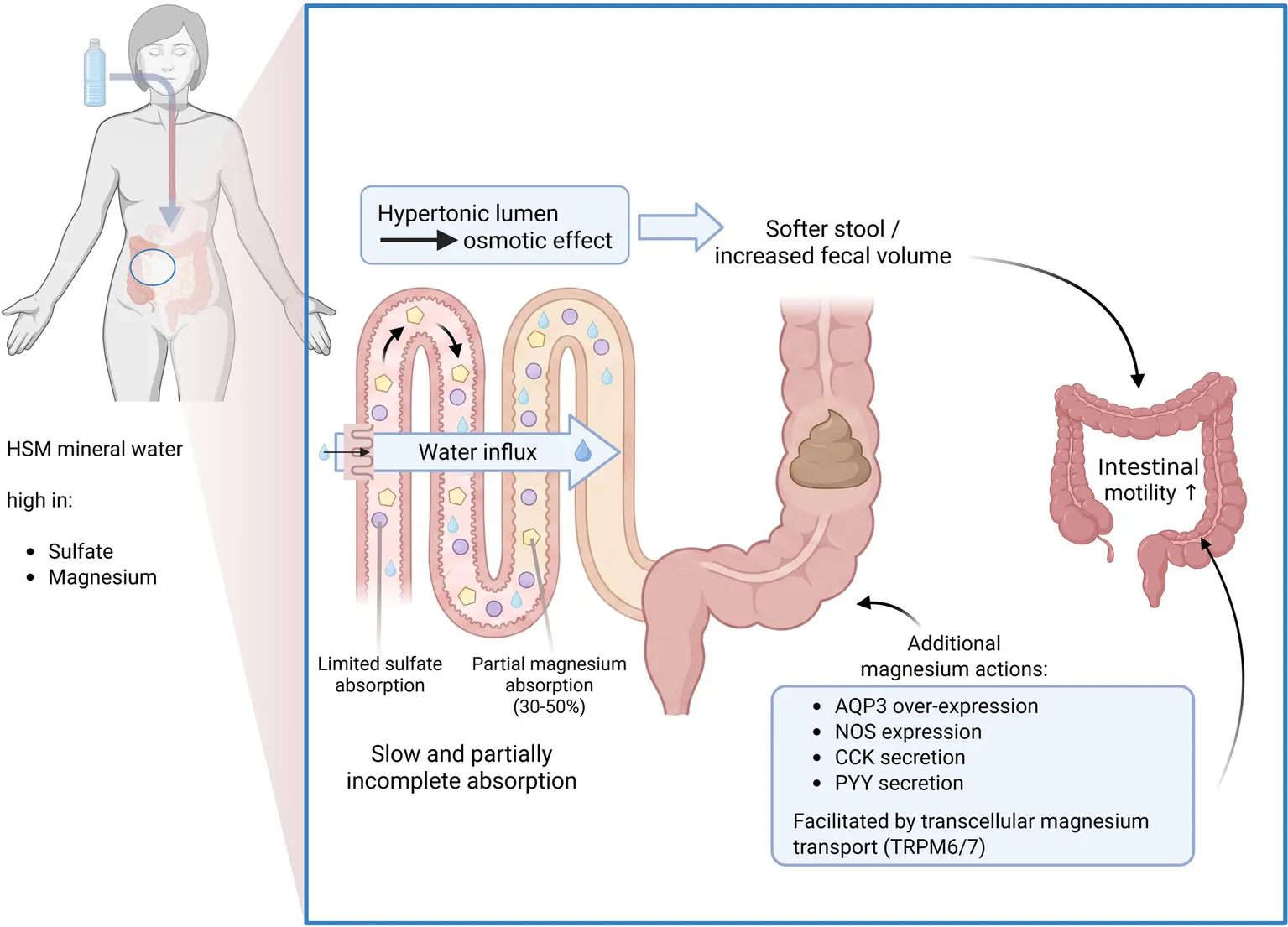
Mechanisms of sulfate- and magnesium-rich mineral water. HSM = High sulfate and magnesium; AQP3 = Aquaporin 3; CCK = Cholecystokinin; NOS = Nitric Oxide Synthase; PYY = Peptide YY; TRPM = Transient Receptor Potential Melastatin - Channel. Created in BioRender. Mansouri. (2026) (36) https://BioRender.com/rhsg32o

### 4.3 Strengths and limitations

It is important to highlight some strengths and limitations. This study constitutes the first intervention study to be conducted entirely online in the field of mineral water for the treatment of chronic constipation. The use of this method entails a number of advantages and disadvantages. It facilitated the collection of data from real-world settings with a broad spectrum of symptom combinations, each to be classified as chronic constipation according to the Rome criteria. However, several limitations need to be considered. First, the self-reporting method may have resulted in an overestimation of constipation-related symptoms (e.g., symptom severity) and actual fluid intake, documented in the screening questionnaire during recruitment process. The bias of differences in fluid intake affecting defecation was mitigated by incorporating a run-in week prior to the intervention weeks. By setting a minimum of fluid intake of 1,500 mL per day in the run-in-week, the likelihood of inadequate fluid intake being the underlying cause of constipation was minimized. In addition, rather than relying on information recalled from memory and recorded in the screening questionnaire, baseline data were derived from the run-in week, including details regarding bowel movements and consistency of stool types. Second, given the characteristic taste of sulfate-rich mineral water, at least some of the participants may have been aware of the assignment to the treatment group. Consequently, it is conceivable that the observed outcomes in the active treatment group may be partly attributable to this awareness. Conversely, the possibility of a placebo effect in the LSM group cannot be ruled out, which could provide a rationale for the observed improvement in symptoms in the control group. This improvement in the control group probably undermined the power of the study and hindered an accurate quantification of the effectiveness of this particular brand of mineral water. Moreover, recruitment did not reach the registered target of 206 participants, which had been based on a conservative target power of 95%. However, the 157 randomized participants exceeded the sample size of 126 required under the conventional 80% power assumption. Therefore, while the deviation from the registered recruitment target should be acknowledged, it is unlikely to represent a substantial limitation with respect to detecting the prespecified effect.

Notwithstanding the aforementioned limitations, it is imperative to acknowledge the several strengths of the study. First, the significant number of participants in this double-blind intervention study, who represented all genders and a wide age range, is a key strength. Second, the inclusion of participants from a variety of geographical locations across Germany and the inclusion of individuals regardless of their symptom combinations contributes to enhancing the generalizability of the results. Third, although conducting an online study could impact data accuracy, it also offers the opportunity to engage different types of interested individuals. Those who might have refused to participate in an in-person study due to the sensitive nature of the data are more likely to participate in an online survey. This also increases the generalizability of the results, as it addresses a broader representative sample of the general population. Fourth, constipation-specific questionnaires have enabled the assessment of specific symptoms and the consideration of constipation-related quality of life.

### 4.4 Summary

In summary, the intake of 1 liter of sulfate- and magnesium-rich mineral water in combination with a total fluid intake of at least 1.5 L/day improved the frequency of bowel movements in individuals with chronic constipation, although the between-group difference at the registered primary endpoint (week three) was not statistically significant. The mineral water consumption was associated with a very good safety profile and a perceived reduction in laxative use. Therefore, sulfate- and magnesium-rich mineral water may be considered a supportive, non-pharmacological dietary measure in individuals suffering from chronic constipation.

## Data Availability

The raw data supporting the conclusions of this article will be made available by the authors, without undue reservation.
