# Supplementary material for: Daily consumption of sulfate- and magnesium-rich mineral water enhances bowel function in adults with chronic constipation: a randomized double-blind real-world trial
Source: Front Nutr. 2026 Sep 24;13:1924028. doi: 10.3389/fnut.2026.1924028 (PMC13645552; doi:10.3389/fnut.2026.1924028)
Supplement: Supplementary file 1 [file Data_Sheet_1.docx]

**Daily Consumption of Sulfate- and Magnesium-Rich Mineral Water Enhances Bowel Function in Adults with Chronic Constipation: A Randomized Double-Blind Real-World Trial**

**Katharina Mansouri^1^, Thierry Hanh^2^, Ronja Meyer^1^, Andreas Hahn^1^***

^1^*Institute of Food and One Health, Leibniz University Hanover, Hanover, Germany*

^2^*Independent Researcher, Paris, France*

*** Correspondence:**
Prof. Dr. Andreas Hahn

hahn@foh.uni-hannover.de

Supplementary Material

# 1 Supplementary Table

*Table S1*: Changes in frequency and consistency from baseline to intervention week two in a subgroup comprising subjects of the second and third tertile of the Rome Score (points 30-59; cat. II + points 60-90; cat. III).

| **Parameter** | **HSM** | **LSM** | **Estimated treatment effect**  (HSM vs. LSM)  (**95% CI)** | ***p*-Value**^a^ |
| --- | --- | --- | --- | --- |
| Frequency* | 1.55 ± 3.32 | 0.36 ± 2.45 | 0.031 (0.005 to 0.057) | 0.022 |
| Consistency** | 0.56 ± 1.57 | 0.52 ± 1.31 | 0.047 (-0.474 to 0.568) | 0.857 |

Data are presented as mean change ± SD on the original scale. Between-group effect estimates, 95% confidence intervals, and p-values are based on analyses of log-transformed data (frequency) or untransformed data (consistency). Positive effect estimates indicate higher values in the HSM group than in the LSM group. HSM = High sulfate and magnesium; LSM = low sulfate and magnesium; SD = standard deviation. ^a^One-way ANOVA.

*n_HSM_=64; n_LSM_=59

** n_HSM_=64; n_LSM_=58

*Table S2*: Changes in frequency and consistency from baseline to intervention week three (ITT).

| **Parameter** | **HSM** | **LSM** | **Estimated treatment effect**  (HSM vs. LSM)  (**95% CI)** | ***p*-Value**^a^ |
| --- | --- | --- | --- | --- |
| Frequency* | 1.03 ± 2.28 | 0.51 ± 3.11 | 0.016 (-0.006 to 0.037) | 0.145 |
| Consistency** | 0.49 ± 1.38 | 0.80 ± 1.44 | -0.304 (-0.764 to 0.155) | 0.193 |

Data are presented as mean change ± SD on the original scale. Between-group effect estimates, 95% confidence intervals, and p-values are based on analyses of log-transformed data (frequency) or untransformed data (consistency). Positive effect estimates indicate higher values in the HSM group than in the LSM group. HSM = High sulfate and magnesium; LSM = low sulfate and magnesium; SD = standard deviation. ^a^One-way ANOVA.

*n_HSM_=78; n_LSM_=70

** n_HSM_=78; n_LSM_=69

*Table S3*: Changes in frequency and consistency from baseline to intervention week two (ITT).

| **Parameter** | **HSM** | **LSM** | **Estimated treatment effect**  (HSM vs. LSM)  (**95% CI)** | ***p*-Value**^a^ |
| --- | --- | --- | --- | --- |
| Frequency* | 1.19 ± 3.15 | 0.30 ± 2.40 | 0.024 (0.001 to 0.047) | 0.045 |
| Consistency** | 0.42 ± 1.47 | 0.49 ± 1.29 | - 0.071 (-0.524 to 0.382) | 0.757 |

Data are presented as mean change ± SD on the original scale. Between-group effect estimates, 95% confidence intervals, and p-values are based on analyses of log-transformed data (frequency) or untransformed data (consistency). Positive effect estimates indicate higher values in the HSM group than in the LSM group. HSM = High sulfate and magnesium; LSM = low sulfate and magnesium; SD = standard deviation. ^a^One-way ANOVA.

*n_HSM_=78; n_LSM_=70

** n_HSM_=78; n_LSM_=69

*Table S4*: Changes in frequency and consistency from baseline to intervention week two in a subgroup comprising subjects of the second and third tertile of the Rome Score (points 30-59; cat. II + points 60-90; cat. III) (ITT).

| **Parameter** | **HSM** | **LSM** | **Estimated treatment effect**  (HSM vs. LSM)  (**95% CI)** | ***p*-Value**^a^ |
| --- | --- | --- | --- | --- |
| Frequency* | 1.40 ± 3.22 | 0.31 ± 2.36 | 0.028 (0.004 to 0.053) | 0.024 |
| Consistency** | 0.51 ± 1.51 | 0.54 ± 1.29 | -0.022 (-0.505 to 0.462) | 0.929 |

Data are presented as mean change ± SD on the original scale. Between-group effect estimates, 95% confidence intervals, and p-values are based on analyses of log-transformed data (frequency) or untransformed data (consistency). Positive effect estimates indicate higher values in the HSM group than in the LSM group. HSM = High sulfate and magnesium; LSM = low sulfate and magnesium; SD = standard deviation. ^a^One-way ANOVA.

*n_HSM_=70; n_LSM_=64

** n_HSM_=70; n_LSM_=63
